# A high serum dehydroepiandrosterone concentration is a predictor of candidates for active surveillance in men with serum prostate-specific antigen < 10 ng/mL

**DOI:** 10.1186/s12885-022-10251-w

**Published:** 2022-11-12

**Authors:** Yasuhide Miyoshi, Takashi Kawahara, Hiroji Uemura

**Affiliations:** 1 Department of Urology, Yokohama City University Graduate School of Medicine, 3-9 Fukuura, Kanazawa-ku, Yokohama, 236-0004 Japan; 2 Present Address: Department of Urology and Renal Transplantation, Yokohama City University Medical Center, 4-57 Urafune-Cho, Minami-Ku, Yokohama, 232-0024 Japan

**Keywords:** Dehydroepiandrosterone, Prostate biopsy, Gleason scores, Liquid chromatography, Biomarker, Active surveillance

## Abstract

**Background:**

There is no consensus on the role of serum dehydroepiandrosterone (DHEA) concentrations in the detection of prostate cancer. This study examined the effectiveness of serum DHEA in predicting candidate patients for active surveillance (AS) prior to prostate biopsy.

**Methods:**

A systematic prostate needle biopsy was performed in 203 men with serum PSA levels of < 10 ng/mL to detect prostate cancer. Serum DHEA concentrations were measured with liquid chromatography-tandem mass spectrometry (LC–MS/MS) just before biopsy. Patient’s age, serum prostate-specific antigen (PSA) levels, prostate volume, and serum DHEA concentrations were compared with pathological findings in multivariate analyses.

**Results:**

The median patient’s age, PSA, serum DHEA concentration and prostate volume were 68 years, 5.5 ng/mL, 1654.7 pg/mL, and 31.2 mL, respectively. In a multivariate analysis, low PSA values, high serum DHEA concentrations, and large prostate volume were significant predictors of the patients with benign prostatic hyperplasia (BPH) or prostate cancer with a Gleason score of ≤ 3 + 4 who are candidate for AS. The DHEA cut-off point for predicting BPH or prostate cancer with a Gleason score of ≤ 3 + 4 was 2188 pg/mL, with a sensitivity, specificity, positive predictive value, and negative predictive value of 33.7%, 96.0%, 98.4%, and 16.9%, respectively.

**Conclusion:**

The study indicated that higher serum DHEA concentrations prior to prostate biopsy might predict the patients with BPH or prostate cancer with a Gleason score ≤ 3 + 4 who are candidate for AS, in men with PSA of < 10 ng/mL.

## Background

Prostate cancer is the second most frequently diagnosed cancer in men and the fifth leading cause of death worldwide [1]. Based on GLOBOCAN 2020 estimates, 1,414,259 new cases of prostate cancer were reported worldwide in 2022. Men in Eastern Asia have lower incidence rate (16.8 per 100,000 people) compared to men in western countries; however, the incidence rate has recently started increasing gradually because of widespread prostate-specific antigen (PSA) screening [1]. PSA screening was shown to be effective in reducing the incidence of prostate cancer deaths by the phase 3 trial [2, 3]. Although PSA screening could improve prostate cancer mortality, PSA screening could lead to overdiagnosis and overtreatment of prostate cancer [3]. In order to avoid overdiagnosis and overtreatment, various methods of detecting non life-threating prostate cancer, namely, indolent cancer, are required. To prevent overtreatment of prostate cancer, active surveillance (AS) or focal therapy have been introduced for low risk (PSA < 10 ng/mL and Gleason score of 6 [Grade group (GG) 1] and cT1-2a) and highly selected intermediate risk (PSA 10–20 or Gleason score 3 + 4 [GG 2] or cT2b) localized prostate cancer patients [4, 5].

Androgens play an important role in the development, growth, and function of the normal prostate [6]. Dehydroepiandrosterone (DHEA) and its sulfated form, DHEA sulfate (DHEAS), are major androgens secreted by the adrenal cortex and are the most abundant circulating steroid hormones in humans [7, 8]. Previous reports showed that DHEA might be associated with the development of prostate cancer [9, 10]; however, the value of serum DHEA levels required for prostate cancer detection is unknown.

In this study, we investigated the correlation between serum DHEA concentrations measured by liquid chromatography-tandem mass spectrometry (LC–MS/MS) in men with PSA levels of < 10 ng/mL prior to an initial prostate biopsy and prostate cancer detection, with the aim of differentiating men with benign prostatic hyperplasia (BPH) or prostate cancer with a Gleason score of ≤ 3 + 4 (≤ GG 2), who have a small risk of progression and candidate for AS, from men with a Gleason score of ≥ 4 + 3 (≥ GG 3).

## Methods

### Patients

An initial prostate needle biopsy was performed in 203 patients with PSA levels of < 10 ng/mL. Patients who were re-biopsied or prescribed finasteride/dutasteride were excluded from this study. This study was retrospective fashion.

### Measurement of serum DHEA concentrations

All blood samples were taken between 09.00 and 15.00 h to minimize the effect of daily DHEA variations. Serum samples were immediately separated from blood and stored at -70 °C until analysis. Serum hormone concentrations were measured with LC–MS/MS, as described by Arai et al. [11]. 100–200 μL serum was used for analysis.

### LC–MS/MS

The detailed procedure for serum DHEA measurement was described previously [11]. serum DHEA concentrations were measured using an API-5000 triple-stage quadrupole mass spectrometer (Applied Biosystems, Foster City, CA, USA), connected to a Shimadzu LC-20 AD pump, SIL HTC autosampler (Kyoto, Japan), and electrospray ionization ion-source device. The columns used were a Capcell Pak SCX UG80 (Shiseido, Tokyo, Japan) pre-column (35 mm × 2 mm internal diameter, particle size 5 μm) and a Cadenza CD-C18 (Imtakt, Kyoto, Japan) analytical column (150 mm × 3 mm internal diameter, particle size 3 μm), which were maintained at 40 °C. The mobile phase consisted of acetonitrile–methanol (50:50, v/v) (solvent A) and 0.1% formic acid (solvent B). For gradient elution, solvents A and B were used at a ratio of 60/40–90/10 between 0 and 5 min and at a ratio of 90/10–100/0 between 5 and 7 min. Solvent A alone was used between 7 and 9 min and solvents A and B at 100/0–60/40 between 9 and 11 min. The flow rate was 0.4 mL/min. The following ESI conditions were used: spray voltage, 3,300 V; collision gas, nitrogen, 45 psi; curtain gas, 11 psi; ion source temperature, 600 °C; and ion polarity, positive.

### Histopathological specimens

The pathological grades of the prostatic tumors were determined using the Gleason grading system [12]. Typical histopathological features using hematoxylin and eosin stain of prostate needle biopsy specimens according to Gleason grading system were obtained from some patients. Histological diagnoses were made by the central pathologist, who was blinded to the serum DHEA concentrations. Immunohistochemical α-methylacyl CoA racemase/P504S staining was performed in specimens that were diagnosed as high-grade prostatic intraepithelial neoplasia or atypical glands; they were classified into BPH or prostate cancer.

### Statistical analyses

Patient characteristics were compared between patients without cancer, those with cancers having Gleason score of ≤ 3 + 4 (≤ GG 2), and those with cancers having Gleason score of ≥ 4 + 3 (≥ GG 3) using the Mann–Whitney U test. All variables were compared using Spearman’s rank correlation coefficient. If some factors were correlated with others, the correlated factors were not analyzed simultaneously because of multicollinearity. Univariate and multivariate logistic regression models were used for detecting the individual factors which predictors of BPH or cancers with a Gleason score of ≤ 3 + 4 (≤ GG 2). Relative risks and 95% confidence intervals (CIs) were derived. All variables were analyzed as continuous variables. The area under the curves (AUCs) was used for setting the cut-off point of the serum DHEA concentration for predicting BPH or Gleason ≤ 3 + 4 (≤ GG 2) prostate cancer. All data were analyzed using the IBM SPSS software, version 26, and the R Stats Package (R Foundation for Statistical Computing, Vienna, Austria).

## Results

**Fig. 1:**
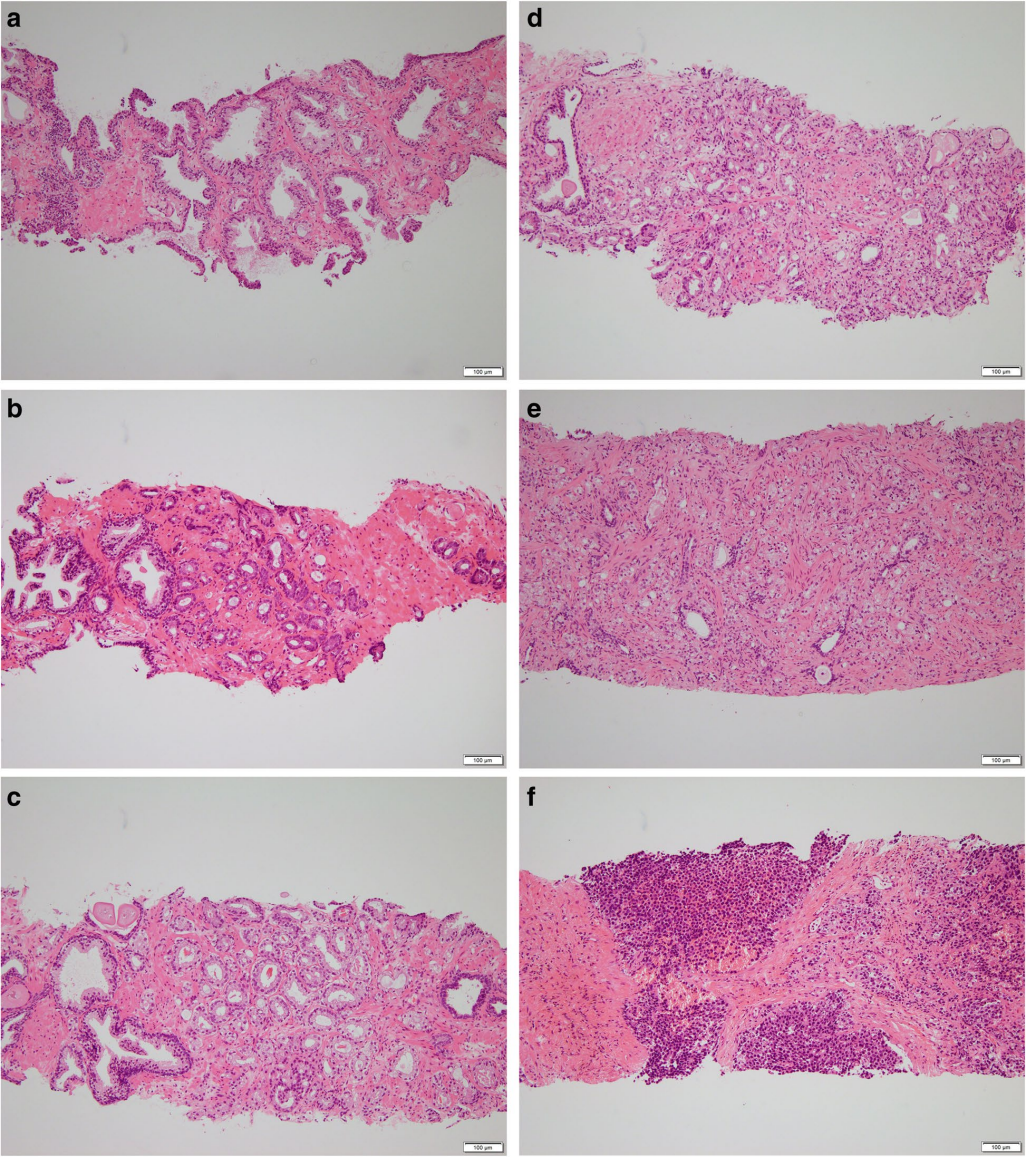
Typical histopathological features of prostate needle biopsy specimens. Hematoxylin and eosin stain. **a** BPH, **b** Adenocarcinoma with GS 3 + 3 (GG 1): Low-grade cancer. Only individual discrete well-formed glands, **c** GS 3 + 4 (GG 2): Intermediate-grade cancer. Predominantly well-formed glands with lesser component of poorly formed/fused/cribriform glands, **d** GS 4 + 3 (GG 3): Intermediate-grade cancer. Predominantly poorly formed/fused/cribriform glands with lesser component of well-formed glands, **e** GS8 (GG 4): High-grade cancer. Only poorly formed/fused/cribriform glands, **f** GS 9–10 (GG 5): High-grade cancer. Lack gland formation (or with necrosis) with or w/o poorly formed/fused/cribriform glands. BPH: benign prostatic hypertrophy; GS: Gleason score; GG: grade group

Relevant patient characteristics are shown in Table 1. BPH was diagnosed in 118 out of 203 patients (58.1%), and prostate cancer was diagnosed in 85 out of 203 (41.9%) patients, including 31 (15.3%) patients with a Gleason score of 6 (GG 1), 29 (14.3%) patients with score of 3 + 4 (GG 2), 7 (3.4%) patients with score of 4 + 3 (GG 3), 9 (4.4%) patients with a score of 8 (GG 4), 9 (4.4%) patients with a score of 9 (GG 5), and 0 with a score of 10 (GG 5). Typical histopathological features of prostate needle biopsy specimens (BPH, adenocarcinoma with GG 1, GG 2, GG 3, GG 4, and GG 5) are shown in Fig. 1.

**Table 1:** Characteristics of patients assessed

Median (range)	Overall (*n* = 203)	Patients with BPH or GGG 1–2 prostate cancer	Patients with GGG 3–5 prostate cancer	*p*-value
		(*n* = 178, 87.7%)	(*n* = 25, 12.3%)	
Median age (range), years	68 (47–81)	67 (47–81)	72 (53–80)	0.004
Median PSA (range), ng/mL	5.5 (1.5–9.9)	5.5 (1.5–9.9)	6.4 (2.7–9.6)	0.027
Median DHEA (range), pg/mL	1654.7 (419.4–8989.3)	1704.3 (419.4–8989.3)	1334.6 (457.4–3563.4)	0.040
Median prostate volume (range), mL	31.2 (8.4–102.3)	31.4 (8.4–102.3)	25.3 (11.5–58.0)	0.035

*BPH* Benign prostatic hypertrophy, *PSA* Prostate-specific antigen, *DHEA* Dehydroepiandrosterone, *GS* Gleason scores

Median age of men with BPH, GG 1–2, and GG 3–5 was 67 (range: 47–80), 67 (range: 51–81), and 72 (53–80) years old, respectively. Median PSA levels of men with BPH, GG 1–2, and GG 3–5 were 5.5 (range: 1.5–9.9), 5.4 (range: 2.8–9.6), and 6.4 (2.7–9.6) ng/ml, respectively. Median DHEA levels of men with BPH, GG 1–2, and GG 3–5 were 1726.7 (range: 422.5–8989.3), 1661.2 (range: 419.4–7053.3), and 1334.6 (457.4–3563.4) pg/ml, respectively.

Having BPH or prostate cancer with a Gleason score of ≤ 3 + 4 (≤ GG 2) was significantly correlated more with a younger age, lower serum PSA concentration, higher serum DHEA concentration, and larger prostate volume, compared to having a Gleason ≥ 4 + 3 cancer (≥ GG 3) (Table 1). Distribution of clinical stage of men with a Gleason score of 6 (GG 1), score of 3 + 4 (GG 2), score of 4 + 3 (GG 3), score of 8 (GG 4) and score of 9–10 (GG 5) were shown in Table 2.

**Table 2:** Correlations between Gleason score (grades group) and clinical stage

	T1cN0M0	T2a-bN0M0	T2cN0M0	T3N0M0	TxN1M0	TxNxM1
GS 6 (GG 1)	17 (54.8%)	7 (22.6%)	7 (22.6%)	0 (0.0%)	0 (0.0%)	0 (0.0%)
GS 3 + 4 (GG 2)	16 (55.2%)	8 (27.6%)	5 (17.2%)	0 (0.0%)	0 (0.0%)	0 (0.0%)
GS 4 + 3 (GG 3)	3 (42.9%)	4 (57.1%)	0 (0.0%)	0 (0.0%)	0 (0.0%)	0 (0.0%)
GS 8 (GG 4)	2 (22.2%)	5 (55.6%)	0 (0.0%)	1 (11.1%)	0 (0.0%)	1 (11.1%)
GS 9–10 (GG5)	3 (33.3%)	3 (33.3%)	1 (11.1%)	2 (22.2%)	0 (0.0%)	0 (0.0%)

*GS* Gleason score, *GG* Grade group

**Fig. 2:**
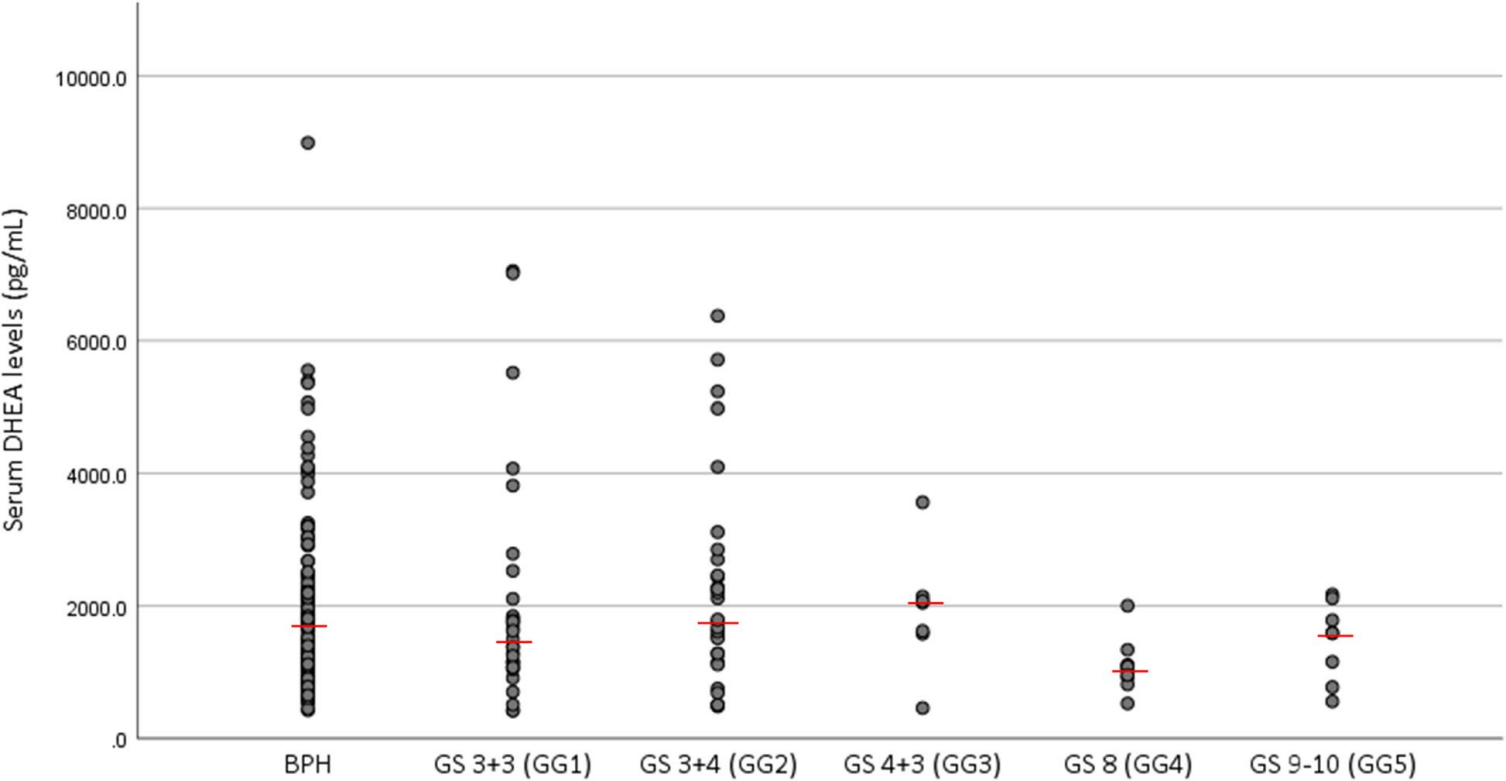
Distribution of serum DHEA levels according to pathological findings. BPH: benign prostatic hypertrophy; GS: Gleason score; GG: grade group; DHEA: dehydroepiandrosterone. The red bar indicates median DHEA levels

The median serum DHEA concentration of the entire cohort was 1654.7 pg/mL (range: 419.4–8989.3 pg/mL). The distribution of serum DHEA levels according to the pathological findings is shown in Fig. 2. There were no strong correlations between serum DHEA concentrations and serum PSA and prostate volume (Table 3).

**Table 3:** Correlations between DHEA concentrations in serum and clinical variables (rs)

	Age	PSA	DHEA	Prostate volume
Age	-	0.19	-0.28	0.19
PSA	0.19	-	0.06	0.25
DHEA	-0.28	0.06	-	0.08
Prostate volume	0.19	0.25	0.08	-

*PSA* Prostate-specific antigen, *DHEA* Dehydroepiandrosterone

**Fig. 3:**
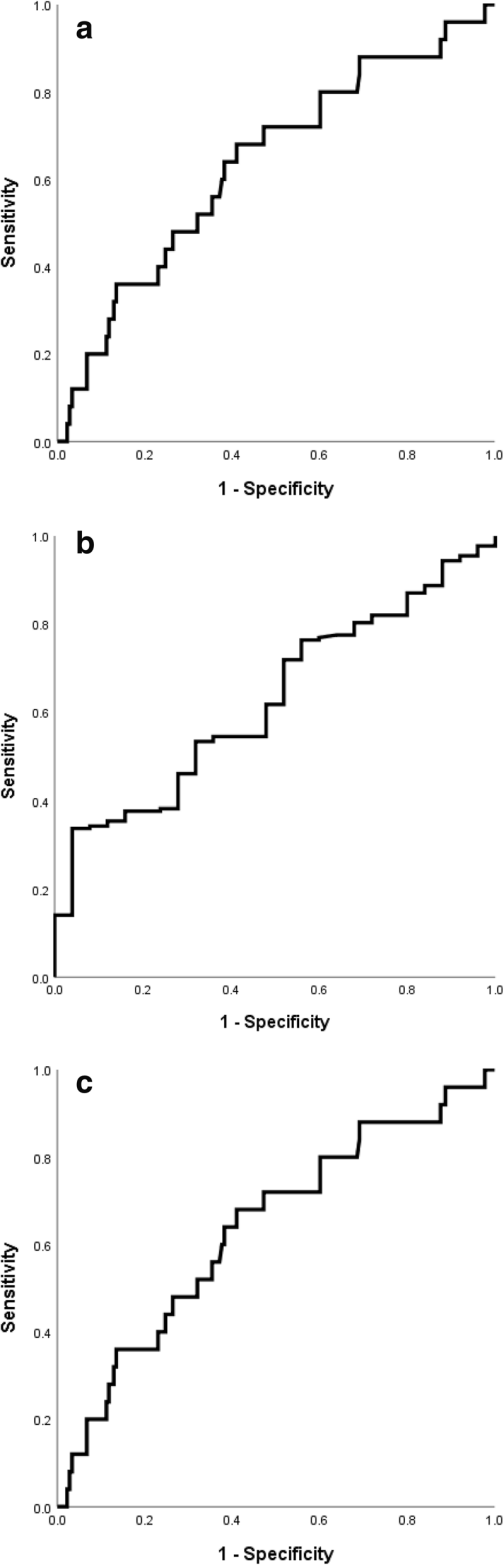
Area under the curve for predicting the probability of BPH or GG 1–2 prostate cancer who have low progression risk. **a** by PSA, **b** by DHEA and **c** by prostate volume. BPH: benign prostatic hypertrophy; GG: grade group; PSA: prostate-specific antigen; DHEA: dehydroepiandrosterone

The AUCs of individual variables for predicting BPH or prostate cancer with a Gleason score of ≤ 3 + 4 (≤ GG 2) are shown in Table 3 and Fig. 3. For predicting BPH or prostate cancer with a Gleason score of ≤ 3 + 4 (≤ GG 2), the AUCs for serum PSA, serum DHEA, and prostate volume were 0.64, 0.63, and 0.63, respectively. In a univariate analysis, low serum PSA level (odds ratio [OR]: 0.77, 95% CI: 0.62–0.97, *p* = 0.027), and high serum DHEA concentrations (DHEA/100, OR: 1.09, 95% CI: 1.00–1.11, *p* = 0.037) were significant predictors of BPH or Gleason score of ≤ 3 + 4 (≤ GG 3) prostate cancer (Table 4). According to the multivariate analysis, a low serum PSA level (OR: 0.71, 95% CI: 0.55–0.90, *p* = 0.005), high serum DHEA concentrations (OR: 1.06, 95% CI: 1.06–1.12, *p* = 0.038), and high prostate volume (OR: 1.04, 95% CI: 1.00–1.08, *p* = 0.038) were significant predictors of BPH or prostate cancer with a Gleason score of ≤ 3 + 4 (≤ GG 2) (Table 4).

**Table 4:** Univariate and multivariate analyses predicting the probability of benign prostatic hypertrophy or grade group 1–2 prostate cancer who have low progression risk

	AUC	95% CI		Univariate analysis				Multivariate analysis			
				OR	95% CI		*p* value	OR	95% CI		*p* value
PSA (ng/mL)	0.64	0.52	0.76	0.77	0.62	0.97	0.027	0.71	0.55	0.90	0.005
DHEA/100 (pg/mL)	0.63	0.52	0.73	1.09	1.00	1.11	0.037	1.06	1.06	1.12	0.038
Prostate volume (mL)	0.63	0.49	0.77	1.04	1.00	1.07	0.079	1.04	1.00	1.08	0.038

*GG* Grade group, *PSA* Prostate-specific antigen, *DHEA* Dehydroepiandrosterone, *OR* Odds ratio, *CI* Confidence interval, *AUC* Area under the curve

The DHEA cut-off point for predicting BPH or prostate cancer with a Gleason score of ≤ 3 + 4 (≤ GG 2) was set at 2188.0 pg/mL, with a sensitivity, specificity, positive predictive value, and negative predictive value of 33.7%, 96.0%, 98.4%, and 16.9%, respectively.

## Discussion

Traditionally, serum androgens including DHEA levels were measured by immunoassays. However, immunoassays for androgens have a lack of precision due to cross reactivity with other steroid hormones. Moreover, low levels of steroid hormones are usually measured in a section of the calibration curve where the variance is high [13]. On the other hands, LC–MS/MS has an advantage in the view of precision in the measurement of androgen levels. LC–MS/MS is over 10 times as sensitive as radioimmunoassay [13]. Moreover, LC–MS/MS also could measure very low levels of steroids in a small amount of specimen. Therefore, today, LC–MS/MS analysis has become the standard method for measuring steroids [13]. The methods of DHEA measurement using LC-MSMS were described in detail by Shibata et al. [13].

Previous studies have shown the correlation between serum DHEA concentration and risk of some solid malignancies including prostate cancer [14–16]. The findings of our study indicate that high pre-biopsy serum DHEA concentrations can predict the patients with BPH or prostate cancer with a Gleason score of ≤ 3 + 4 (≤ GG 2), who are candidate for AS because of small progression risk. The role of high serum DHEA concentrations in the prevention of the development and progression of prostate cancer is unclear. Our results are consistent with the school of thought that serum androgen concentrations correlate with prostate cancer aggressiveness. Garnham et al., were the first to report a correlation between and prostate cancer and DHEA or DHEAS levels measured using gas–liquid chromatography [17]. There are several retrospective reports on the correlation between serum DHEA concentrations and prostate cancer prognosis. We previously reported that low serum DHEA concentrations examined by LC–MS/MS were correlated with higher Gleason scores (a poor prognostic marker) and advanced clinical stage in 196 patients with hormone-naïve prostate cancer (HNPC), retrospectively [18]. Ryan et al., also reported the correlation between baseline DHEAS and overall survival in an ad hoc analysis of a randomized phase III, COU-AA-301 trial, which is a multinational, randomized, double-blind study of abiraterone acetate plus prednisone versus placebo plus prednisone in patients with metastatic castration-resistant prostate cancer (mCRPC) after receiving docetaxel. DHEAS was examined by LC–MS/MS. They demonstrated that lower baseline serum DHEAS concentrations were predictive biomarkers of poorer overall survival in patients treated with both abiraterone acetate plus prednisone and placebo plus prednisone [19].

Suzuki et al., also retrospectively evaluated the correlation between baseline serum DHEAS and oncological outcome after abiraterone acetate plus prednisolone treatment in 28 mCRPC patients [20]. They concluded that patients with higher baseline serum DHEAS levels had a significantly longer PSA progression-free survival compared to those with lower baseline DHEAS. Yano et al., also reported that low baseline serum DHEAS concentrations are predictors of a poor response to androgen deprivation therapy for HNPC [21]. Furthermore, the time to CRPC from androgen deprivation therapy in the patients with higher DHEA levels was longer compared to those with lower DHEA levels (*p* = 0.015).

Severi et al., reported the correlation between serum DHEAS levels and prostate cancer risk in their prospective large cohort study that included 17,049 men [22]. They used a case-cohort design, including 524 cases diagnosed during a mean period of 8.7 years of follow-up and a randomly sampled subcohort of 1,859 men. They found that there was no correlation between serum DHEAS levels and prostate cancer detection risk. However, in the detection of aggressive cancer (Gleason score of ≥ 8 or clinical stage was advanced [T4 or N + or M +]), there was significant risk correlation between serum DHEAS levels and aggressive prostate cancer detection risk. Hazard ratio for quartile IV, III, II compared to quartile I (reference) were 0.38 (95% CI 0.15–0.95), 0.54 (95% CI 0.29–1.02), and 0.53 (95% CI 0.31–0.92), respectively (*p* = 0.007). Those results are consistent with the result of our study.

As mentioned above, prognosis tends to be unfavorable in prostate cancer patients with lower baseline serum DHEA levels than in those with higher DHEA levels; however, no confirmatory prospective result has been obtained. On the other hand, there are several negative reports on prostate cancer detection and DHEA. In the age- and race-matched control study by Comstock et al., DHEA and DHEAS were not important risk factors for prostate cancer [23].

Corder et al., also reported that DHEAS was not a predictor of prostate cancer [24]. Roddam et al., performed a meta-analysis of 18 prospective studies including 3886 men with incident prostate cancer and 6438 control subjects [25]. They found that there was no association between the risk of prostate cancer and serum concentrations of DHEAS.

Even in our study, DHEA levels could not be used to detect prostate cancer among men with PSA of ≤ 10 ng/mL although DHEA levels can be used to predict BPH or prostate cancer with a Gleason score of ≤ 3 + 4 (≤ GG 2).

For selecting prostate cancer treatments, pathological findings according to Gleason grading system are crucial. Men with PSA of ≤ 10 ng/mL and biopsy Gleason 6 (GG 1) prostate cancers usually could be managed through AS, not with curative treatment, such as prostatectomy and radiotherapy because Gleason 6 (GG 1) cancers have extremely low progression risk [26]. Highly selected Gleason 3 + 4 (GG 2) cancers have also low progression risks [26, 27]. Klotz et al., reported that AS should be recommended for both Gleason 6 (GG 1) and highly selected patients with Gleason 3 + 4 (GG 2) cancers. From our results, high serum DHEA levels prior to prostate biopsy could detect the patients with BPH or prostate cancer with a Gleason score of ≤ 3 + 4 (≤ GG 2). When selecting the treatment options for men with low progression risks, serum DHEA levels could be useful in determining good candidates for minimum invasive treatment, such as AS or focal therapy, although prospective confirmatory studies will be needed.

The Prostate Health Index (PHI) [28] and 4 K scores [29] have been shown to be strong predictors of high-grade prostate cancer. Therefore, we found that serum DHEA alone could not aid in deciding whether to perform a biopsy. We propose that in addition to other clinical factors, such as age, PSA, prostate volume, PHI and 4 K scores, the pre-biopsy serum DHEA concentration measured with LC–MS/MS may be a useful biomarker for predicting BPH or low progression risks prostate cancer.

This study has several limitations. First, it was a small retrospective study. Second, true Gleason scores derived from prostatectomies were not determined, even though Gleason score up-grading between prostate biopsies and prostatectomies reportedly occurs in almost 30% of cases [30]. Further prospective studies with larger numbers of patients and longer follow-up durations are warranted to confirm the validity of our results. Finally, we did not assess the patients’ outcomes and prognoses. Although there were several study limitations, our study demonstrated that higher serum DHEA concentrations might predict the patients with BPH or prostate cancer with a Gleason score of ≤ 3 + 4 (≤ GG 2) in men with PSA concentrations of < 10 ng/mL. Future studies should be conducted to evaluate both the association of serum DHEA with cancer aggressiveness and cancer prognosis.

## Conclusion

Our study demonstrated that higher serum DHEA concentration predicts benign prostatic hyperplasia or prostate cancer with Gleason score of ≤ 3 + 4 (≤ GG 2) in men with PSA of < 10 ng/mL. Future studies should be conducted to evaluate both the association of serum DHEA with cancer aggressiveness and cancer prognosis.

## Data Availability

The data presented in this study are available on request from the corresponding author. The data are not publicly available.

## Abbreviations

PSA: Prostate-specific antigen
AS: Active surveillance
GG: Grade group
DHEA: Dehydroepiandrosterone
DHEAS: Dehydroepiandrosterone sulfate
LC–MS/MS: Liquid chromatography-tandem mass spectrometry
BPH: Benign prostatic hyperplasia
CIs: Confidence intervals
AUCs: Area under the curves
OR: Odds ratio
HNPC: Hormone-naïve prostate cancer
mCRPC: Metastatic castration-resistant prostate cancer
